# Traditional Herbal Formula *Banhasasim-tang* Exerts Anti-Inflammatory Effects in RAW 264.7 Macrophages and HaCaT Keratinocytes

**DOI:** 10.1155/2015/728380

**Published:** 2015-03-09

**Authors:** Seong Eun Jin, Hye-Sun Lim, Yeji Kim, Chang-Seob Seo, Sae-Rom Yoo, Hyeun-Kyoo Shin, Soo-Jin Jeong

**Affiliations:** ^1^Herbal Medicine Formulation Research Group, Herbal Medicine Research Division, Korea Institute of Oriental Medicine, Daejeon 305-811, Republic of Korea; ^2^Division of Allergy and Chronic Respiratory Diseases, Center for Biomedical Sciences, Korea National Institute of Health, Chungcheongbuk-do 361-951, Republic of Korea

## Abstract

*Banhasasim-tang* (BHSST) is a Korean traditional herbal formula comprising eight medicinal herbs. The aim of the present study was to investigate the anti-inflammatory effect of BHSST using macrophage and keratinocyte cell lines. First, we evaluated the effects of BHSST on inflammatory mediator and cytokine production in lipopolysaccharide- (LPS-) stimulated RAW 264.7 macrophages. BHSST markedly inhibited the production of nitric oxide (NO), prostaglandin E_2_ (PGE_2_), and interleukin- (IL-) 6. BHSST significantly suppressed the protein expression of toll-like receptor 4 (TLR4) and phosphorylated nuclear factor-kappa B (NF-*κ*B) p65 in RAW 264.7 cells. Second, we examined whether BHSST influences the production of chemokines and STAT1 phosphorylation in tumor necrosis factor-*α*/interferon-*γ* TI-stimulated HaCaT keratinocytes. BHSST significantly suppressed the production of RANTES/CCL5, TARC/CCL17, MDC/CCL22, and IL-8 in TI-stimulated HaCaT cells. BHSST also suppressed TI-induced phosphorylation of STAT1 in HaCaT cells. These results suggest that BHSST may be useful as an anti-inflammatory agent, especially for inflammatory skin diseases.

## 1. Introduction

Inflammation is a protective response to pathogens such as bacteria, viruses, and parasites. Coordinate regulation of inflammatory mediator and cytokine release is necessary for optimal host defense. Nitric oxide (NO) and prostaglandin E_2_ (PGE_2_), important inflammatory mediators, are secreted by activated immune cells, such as macrophages. Under pathological conditions, NO production is increased by inducible NOS (iNOS) and subsequently leads to cytotoxicity and tissue damage. PGE_2_ is produced at inflammatory sites by cyclooxygenase-2 (COX-2), which is therefore implicated as an important mediator in the processes of inflammation. In addition, tumor necrosis factor-*α* (TNF-*α*) and interleukin- (IL-) 6 are proinflammatory cytokines that act as signaling molecules for immune cells and coordinate inflammatory responses.

Toll-like receptors (TLRs) mediate signal transduction cascades that ultimately promote the nuclear translocation of nuclear factor-kappa B (NF-*κ*B). In particular, TLR4 is a pattern recognition receptor for lipopolysaccharide (LPS) [1]. NF-*κ*B signaling is considered a pivotal mechanism for the regulation of immune and inflammatory responses by controlling the transcription of inflammatory cytokines [2].

However, overproduction of these inflammatory mediators and cytokines can result in various pathological conditions [3, 4]. In particular, atopic dermatitis- (AD-) like inflammatory skin diseases are aggravated by inflammatory mediators and cytokines in chronic states of inflammation [5]. Migration of inflammatory cells into lesioned skin is regulated by inflammatory chemokines, a group of small cytokines produced by various cell types. In particular, Th2-type cell-specific chemokines, such as regulated on activation, normal T-cell expressed and secreted (RANTES/CCL5), thymus- and activation-regulated chemokine (TARC/CCL17) and macrophage-derived chemokine (MDC/CCL22), are produced from keratinocytes, and may be effective targets for the treatment of inflammatory skin diseases [6]. In addition, IL-8 is also an important mediator in inflammatory skin diseases [7].

Traditional herbal formulas have been widely used over several thousand years to prevent and treat various diseases in East Asia, including Korea, China, and Japan [8]. Among them,* Banhasasim-tang* (BHSST,* Hange-shashin-to*, and* Ban xia xie xin tang*), comprising eight medicinal herbs (Table 1), has been used for treating gastritis, diarrhea and gastric ulcer [9, 10]. Despite previous reports, there have been no reported studies on the effects or action mechanisms of BHSST on inflammatory responses.

In this study, we investigated the anti-inflammatory effects of BHSST in RAW 264.7 macrophages and HaCaT keratinocytes* in vitro*.

## 2. Materials and Methods

### 2.1. Plant Materials

The eight medicinal herbs forming BHSST were purchased from Kwangmyungdang (Ulsan, Korea). The origin of eight medicinal herbs was confirmed taxonomically by Professor Je-Hyun Lee, Dongguk University, Gyeongju, Republic of Korea. A voucher specimen (2012–KE38–1~KE38–8) has been deposited at the Herbal Medicine Formulation Research Group, Korea Institute of Oriental Medicine.

### 2.2. Chemicals and Reagents

**Figure 1:**
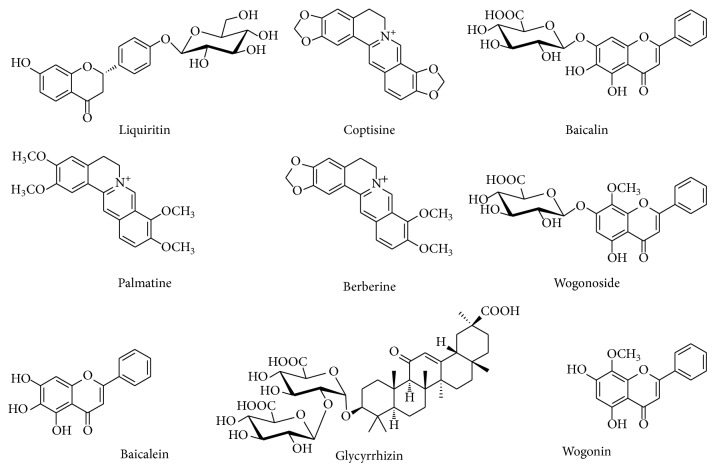
Chemical structures of the nine marker compounds in* Banhasasim-tang*.

Liquiritin, coptisine, baicalin, palmatine, berberine, glycyrrhizin, and wogonin were purchased from Wako Chemicals (Osaka, Japan). Wogonoside was purchased from Tauto Biotech (Shanghai, China) and baicalein was purchased from Sigma-Aldrich (St. Louis, MO, USA). The purity of each component was determined ≥98% by HPLC analysis. The chemical structures of the nine marker compounds are shown in Figure 1. HPLC-grade reagents, methanol, acetonitrile, and water were obtained from J.T. Baker (Phillipsburg, NJ, USA). Trifluoroacetic acid, lipopolysaccharide (LPS) from* Escherichia coli*,* N* ^*G*^-methyl-l-arginine (l-NMMA), indomethacin, and CelLytic MT Cell Lysis Reagent were purchased from Sigma-Aldrich. Dulbecco's modified Eagle's medium (DMEM), fetal bovine serum (FBS), penicillin-streptomycin, and phosphate-buffered saline (PBS) were obtained from Gibco BRL (Grand Island, CA, USA). The Cell Counting Kit-8 (CCK-8) used was a product of Dojindo (Kumamoto, Japan). Griess reagent and PGE_2_ enzyme-linked immunosorbent assay (ELISA) kit were purchased from Promega Corporation (Madison, WI, USA) and Cayman Chemical Co. (Ann Arbor, MI, USA), respectively. Mouse TNF-*α* and IL-6 ELISA kits and Trizol reagent were obtained from Invitrogen (Camarillo, CA, USA). Human recombinant TNF-*α* and interferon-*γ* (IFN-*γ*), and human chemokine (RANTES, TARC, MDC, and IL-8) ELISA kits were purchased from R&D Systems Inc. (Minneapolis, MN, USA). An iScript cDNA Synthesis kit and Protein Assay Dye Reagent Concentrate were purchased from Bio-Rad Laboratories (Hercules, CA, USA). The enhanced chemiluminescence (ECL) kit used was a product of Thermo Fisher Scientific (Waltham, MA, USA).

### 2.3. Preparation of BHSST Decoction

BHSST comprised eight herbs (Table 1, total weight = 5.0 kg, about 133.3 times the composition of a single dose) and was extracted in distilled water at 100°C for 2 h under pressure (98 kPa) using an electric extractor (COSMOS-660; Kyungseo Machine Co., Incheon, Korea). The extract solution was filtered using a standard sieve (No. 270, 53 *μ*m) and freeze-dried. The yield of extract was 14.99% (749.5 g). For quantitative analysis, lyophilized BHSST extract (200 mg) was dissolved in 70% methanol (20 mL) and mixed. Subsequently, the solution was diluted 10-fold with 70% methanol for quantitative analysis of baicalin and wogonoside. The solution was filtered through a SmartPor GHP syring filter (0.2 *μ*m pore size, Woongki Science, Seoul, Korea) before injection into the HPLC system.

### 2.4. Quantitative Analysis of the Marker Constituents in BHSST

The quantitative determination was performed with a Shimadzu Prominence LC-20A series HPLC system (Kyoto, Japan) consisting of a solvent delivery unit (LC-20AT), on-line degasser (DGU-20A_3_), column oven (CTO-20A), auto sample injector (SIL-20AC), and photodiode array detector (SPD-M20A). Data were collected and processed using LCsolution software (version 1.24; Shimadzu, Kyoto, Japan). The marker compounds were separated on a Gemini C_18_ column (250 mm × 4.6 mm; particle size 5 *μ*m; Phenomenex, Torrance, CA, USA) maintained at 40°C. The mobile phases consisted of 0.1% (v/v) trifluoroacetic acid in distilled water (A) and acetonitrile (B). The gradient flow was as follows: 10–60% B for 0–30 min, 60–100% B for 30–40 min, 100% B for 40–45 min, and 100–10% B for 45–50 min. The analysis was conducted at a flow-rate of 1.0 mL/min with PDA detection at 254 nm (glycyrrhizin), 275 nm (liquiritin, baicalin, wogonoside, baicalein, and wogonin), and 350 nm (coptisine, palmatine, and berberine). The injection volume was 10 *μ*L.

### 2.5. Cell Culture

The murine macrophage RAW 264.7 cells and human keratinocyte HaCaT cells were obtained from the American Type Culture Collection (Rockville, MD, USA) and CLS Cell Lines Service (Eppelheim, Baden-Württemberg, Germany), respectively. RAW 264.7 cells were cultured in DMEM, which was supplemented with 5.5% heat-inactivated FBS, penicillin (100 U/mL) and streptomycin (100 *μ*g/mL) in a 5% CO_2_ incubator at 37°C. HaCaT cells were cultured in DMEM supplemented with 10% heat-inactivated FBS, penicillin (100 U/mL), and streptomycin (100 *μ*g/mL) in a 5% CO_2_ incubator at 37°C.

### 2.6. Cytotoxicity Assay

Cell viability was assessed using a CCK-8 kit according to the manufacturer's instructions. RAW 264.7 cells (3 × 10^3^ cells/well) and HaCaT cells (1 × 10^3^ cells/well) were incubated in 96-well plates with various concentrations of BHSST in the absence or presence of respective LPS (1 *μ*g/mL) and TNF-*α* and IFN-*γ* (TI, each 10 ng/mL) for 24 h. The CCK-8 reagent was added to each well, followed by incubation for an additional 4 h. Absorbance by well contents was measured at 450 nm using a Benchmark plus microplate reader (Bio-Rad Laboratories). The percentage of viable cells was calculated using the following formula: cell viability (%) = (mean absorbance in test wells/mean absorbance in control wells) × 100.

### 2.7. Measurement of Inflammatory Mediator Levels

RAW 264.7 cells (2.5 × 10^5^ cells/well) were cultured in 48-well plates. After reaching confluence, the cells were treated with various concentrations of BHSST for 4 h, and then stimulated with 1 *μ*g/mL of LPS for 20 h. The culture supernatants were used to determine levels of secreted nitrite, PGE_2_, TNF-*α* and IL-6. The nitrite production was measured by Griess reagent as an indicator of NO, and the PGE_2_, TNF-*α*, and IL-6 production were determined using ELISA kits. l-NMMA and indomethacin were used as positive controls for NO and PGE_2_, respectively.

### 2.8. Measurement of Chemokine Production

HaCaT cells (1 × 10^6^ cells/well) were cultured in 6-well plates. After reaching confluence, the cells were washed and treated with various concentrations of BHSST in 1 mL of serum-free medium that contained TI (each 10 ng/mL) for 24 h. The supernatants were harvested, and production of RANTES, TARC, MDC, and IL-8 were determined using ELISA kits.

### 2.9. Western Blotting

To determine the effect of BHSST on expression of COX-2, TLR4, and phosphorylated NF-*κ*B in RAW 264.7 cells, the cells were pretreated with various concentrations of BHSST for 2 h, and then stimulated with LPS (1 *μ*g/mL) for 14 h and 30 min, respectively. HaCaT cells were treated with TI in the presence or absence of various concentrations of BHSST for 30 min to investigate the influence of BHSST on STAT1 activation. The cells were collected by centrifugation and washed twice with PBS. Proteins of whole cells were extracted using CelLytic MT Cell Lysis Reagent containing protease inhibitors. Nuclear proteins were extracted using NE-PER Nuclear and Cytoplasmic Extraction Reagents (Thermo Fisher Scientific Inc., Waltham, MA, USA) according to the manufacturer's protocol. The protein concentration was determined using a Protein Assay Dye Reagent Concentrate according to the manufacturer's instructions. Total proteins (15–20 *μ*g) were resolved by 10% sodium dodecyl sulfate-polyacrylamide gel electrophoresis (SDS-PAGE) and transferred to PVDF membranes. The membranes were immediately blocked with 5% skim milk in Tris-buffered saline containing 0.1% Tween-20 (pH 7.4) (TBST), followed by incubation with primary antibodies against COX-2 (Santa Cruz Biotechnology, Santa Cruz, CA, USA), TLR4 (Santa Cruz Biotechnology), phospho-NF-*κ*B p65 (Cell Signaling Tech., Danvers, MA, USA), STAT-1 (Abcam, Cambridge, MA, USA), phospho-STAT1 (Abcam), and *β*-actin (Cell Signaling Tech., Danvers, MA, USA) at 4°C overnight. After washing three times with TBST, the membranes were incubated with a horseradish peroxidase- (HRP-) conjugated secondary antibody (Jackson Immuno Research, PA, USA) for 1 h at room temperature. The membranes were washed three times with TBST, and then developed using an ECL kit. The membranes were photographed using a ChemiDoc XRS^+^ imaging system (Bio-Rad Laboratories).

### 2.10. Immunofluorescence Staining

Cells were seeded onto glass coverslips and incubated with TI (each 10 ng/mL) in the absence or presence of BHSST (500 *μ*g/mL) for 30 min. The cells were fixed in 4% paraformaldehyde and 100% acetone, blocked in 0.5% bovine serum albumin, and incubated with anti-STAT1 antibody (Cell Signaling Tech.) for 1 h at room temperature. Then, FITC-conjugated anti-rabbit immunoglobulin G (IgG) antibody (Invitrogen, Carlsbad, CA, USA) was used as a secondary antibody. The immunostained cells were mounted with medium containing DAPI and visualized by use of an Olympus FLUOVIEW FV10i confocal microscope (Tokyo, Japan).

### 2.11. Statistics

All data are presented as the mean ± SEM. One-way analysis of variance was used to detect significant differences between the control and treatment groups. Dunnett's test was used for multiple comparisons. The differences were considered significant at *P* < 0.05 and *P* < 0.01.

## 3. Results

### 3.1. Quantitative Determination of the Nine Compounds in BHSST

**Figure 2:**
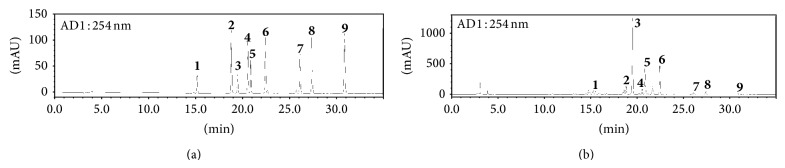
HPLC chromatograms of the standard mixture (a) and* Banhasasim-tang* extract (b) at 254 nm. Liquiritin (**1**), coptisine (**2**), baicalin (**3**), palmatine (**4**), berberine (**5**), wogonoside (**6**), baicalein (**7**), glycyrrhizin (**8**), and wogonin (**9**).

The established HPLC analytical method was applied for the simultaneous quantification of nine compounds in BHSST. The typical chromatogram patterns for standard compounds and the BHSST decoction are shown in Figure 2. The retention times of the liquiritin, coptisine, baicalin, palmatine, berberine, wogonoside, baicalein, glycyrrhizin, and wogonin were detected at approximately 15.14, 18.80, 19.45, 20.54, 20.84, 22.41, 26.10, 27.36, and 30.85 min, respectively. The concentrations of the nine components were 0.977–48.340 mg/g and the results are summarized in Table 2.

### 3.2. Effects of BHSST on Inflammatory Mediators in RAW 264.7 Cells

**Figure 3:**
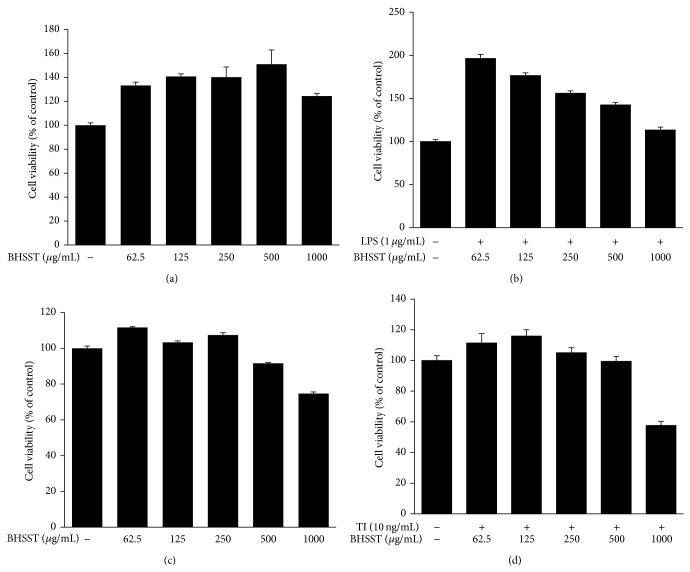
Cytotoxic effects of* Banhasasim-tang* (BHSST) in RAW 264.7 cells and HaCaT cells. RAW 264.7 cells (a) and HaCaT cells (c) were seeded in to 96-well plates and treated with various concentrations of BHSST for 24 h. (b) RAW 264.7 cells were cotreated with various concentrations of BHSST, and LPS (1 *μ*g/mL) for 24 h. (d) HaCaT cells were cotreated with various concentrations of BHSST, and TNF-*α* and IFN-*γ* (each 10 ng/mL, TI) for 24 h. Cell viability was assessed using a CCK-8 kit. The values are expressed as mean ± SEM of three independent experiments.

**Figure 4:**
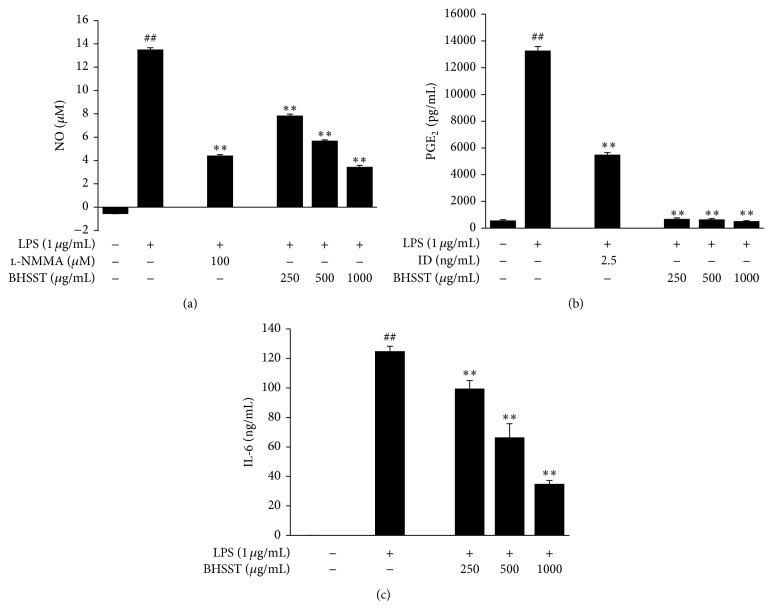
Effects of* Banhasasim-tang* (BHSST) on production of inflammatory mediators and cytokine in RAW 264.7 cells. Cells were pretreated with BHSST (250, 500 or 1000 *μ*g/mL) for 4 h and then stimulated with LPS (1 *μ*g/mL) for 20 h. NO production was measured in the culture supernatant with the Griess reaction (a). The levels of PGE_2_ (b) and IL-6 (c) released into the culture medium were assessed using commercially available ELISA kits. l-NMMA and indomethacin (ID) were used as positive controls for NO and PGE_2_, respectively. Values are expressed as mean ± SEM of three independent experiments. Each bar represents the mean of three independent experiments. ^##^ *P* < 0.01 versus vehicle control cells; and ^**^ *P* < 0.01 versus LPS-treated cells.

The cytotoxicity of BHSST in the absence or presence of LPS (1 *μ*g/mL) for 24 h was determined in RAW 264.7 cells (Figures 3(a) and 3(b)), and subsequent experiments were performed at nontoxic concentrations. To investigate the anti-inflammatory effect of BHSST, we measured NO, PGE_2_, and IL-6 levels in LPS-stimulated RAW 264.7 cells. As shown in Figure 4, LPS-treated RAW 264.7 cells significantly increased NO, PGE_2_, TNF-*α* and IL-6 levels compared with the vehicle-treated cells (*P* < 0.01). By contrast, BHSST (250, 500 or 1000 *μ*g/mL) suppressed LPS-induced NO, PGE_2_, and IL-6 production in a dose-dependent manner compared with cells treated with LPS alone (*P* < 0.01) (Figure 4). However, BHSST had no effect on LPS-induced TNF-*α* production in RAW 264.7 cells (data not shown). Positive controls using l-NMMA and indomethacin showed significant decreases in LPS-stimulated NO and PGE_2_ production, respectively (Figures 4(a) and 4(b)).

### 3.3. Effects of BHSST on Expression of COX-2, TLR4, and Phosphorylated NF-*κ*B in RAW 264.7 Cells

**Figure 5:**
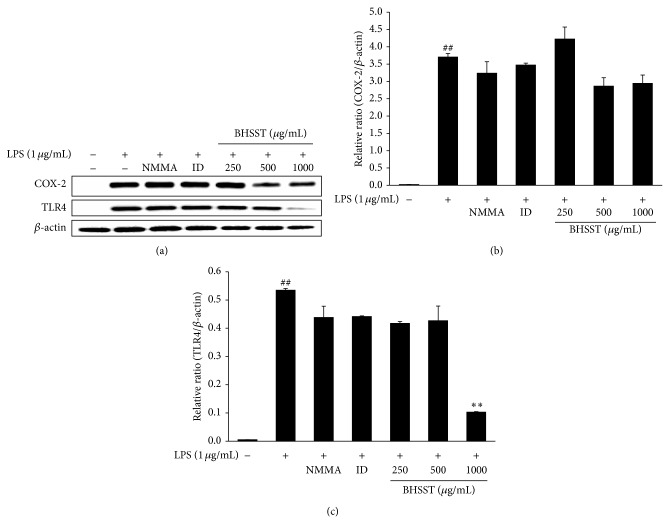
Effects of* Banhasasim-tang* (BHSST) on COX-2 and TLR4 expression in RAW 264.7 cells. Cells were pretreated with BHSST (250, 500 or 1000 *μ*g/mL) for 2 h and then stimulated with LPS (1 *μ*g/mL) for 14 h. (a) Protein expression of COX-2 and TLR4 was analyzed by Western blotting. The relative abundance of protein was calculated as COX-2/*β*-actin ratio (b) and TLR4/*β*-actin ratio (c). Values are expressed as mean ± SEM of three independent experiments. ^##^ *P* < 0.01 versus vehicle control cells; and ^**^ *P* < 0.01 versus LPS-treated cells.

**Figure 6:**
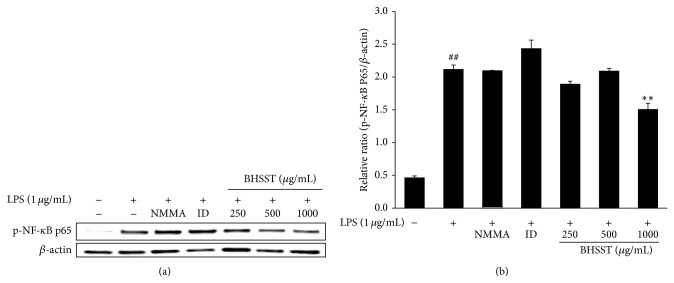
Effect of* Banhasasim-tang* (BHSST) on NF-*κ*B activation in RAW 264.7 cells. Cells were pretreated with BHSST (250, 500 or 1000 *μ*g/mL) for 2 h and then stimulated with LPS (1 *μ*g/mL) for 30 min. (a) Protein expression of phospho-NF-*κ*B p65 was analyzed by Western blotting. The relative abundance of protein was calculated as phospho-NF-*κ*B p65/*β*-actin ratio. Values are expressed as mean ± SEM of three independent experiments. ^##^ *P* < 0.01 versus vehicle control cells; and ^**^ *P* < 0.01 versus LPS-treated cells.

As shown in Figure 5, LPS markedly induced expression of COX-2 and TLR4 compared with the vehicle in RAW 264.7 cells. BHSST had no significantly inhibition on LPS-stimulated COX-2 expression (Figures 5(a) and 5(b)). By contrast, BHSST reduced LPS-stimulated TLR4 expression at a dose of 1000 *μ*g/mL (*P* < 0.01) (Figures 5(a) and 5(c)). To determine whether BHSST mediates the inhibition of inflammatory responses through NF-*κ*B pathway, phosphorylation of NF-*κ*B p65 was analyzed by Western blotting. As shown in Figure 6, phosphorylation of NF-*κ*B p65 was increased by LPS stimulation. By contrast, BHSST significantly suppressed LPS-stimulated phosphorylation of NF-*κ*B p65 at a dose of 1000 *μ*g/mL (*P* < 0.01).

### 3.4. Effects of BHSST on Chemokine Production in HaCaT Cells

**Figure 7:**
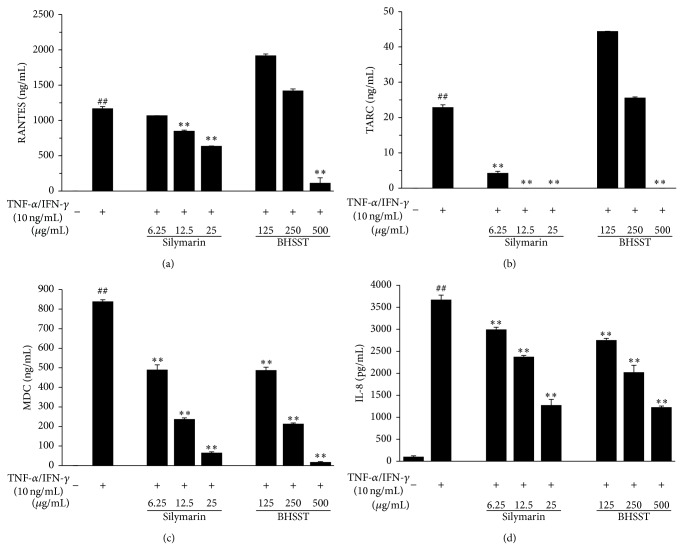
Effects of* Banhasasim-tang* (BHSST) on production of chemokines in HaCaT cells. Production of RANTES (a), TARC (b), MDC (c), and IL-8 (d) were measured using the culture supernatant. Cells were cotreated with BHSST (125, 250, or 500 *μ*g/mL), and TNF-*α* and IFN-*γ* (TI, each 10 ng/mL) for 24 h. Silymarin (6.25, 12.5, or 25 *μ*g/mL) was used as a positive control. Values are expressed as mean ± SEM of three independent experiments. ^##^ *P* < 0.01 versus vehicle control cells; and ^**^ *P* < 0.01 versus TI-treated cells.

The cytotoxicity of BHSST in the absence or presence of TI (each 10 ng/mL) for 24 h was determined in HaCaT cells (Figures 3(c) and 3(d)), and subsequent experiments were performed at nontoxic concentrations. To investigate the inhibitory effect of BHSST on TI-induced production of RANTES, TARC, MDC, and IL-8, HaCaT cells were cotreated with BHSST (125, 250, or 500 *μ*g/mL) and TI for 24 h. The production of RANTES, TARC, MDC, and IL-8 was measured by ELISA. The TI stimulation significantly increased production of RANTES, TARC, MDC, and IL-8 compared with the vehicle in HaCaT cells (Figures 7(a)–7(d)). By contrast, BHSST suppressed TI-stimulated RANTES and TARC production at a dose of 500 *μ*g/mL (*P* < 0.01) (Figures 7(a) and 7(b)). The increased MDC and IL-8 production brought about by TI stimulation were significantly decreased by BHSST in a dose-dependent manner (Figures 7(c) and 7(d)). A positive control, silymarin showed a significant decrease in TI-stimulated chemokine production.

### 3.5. Effects of BHSST on STAT1 Phosphorylation

**Figure 8:**
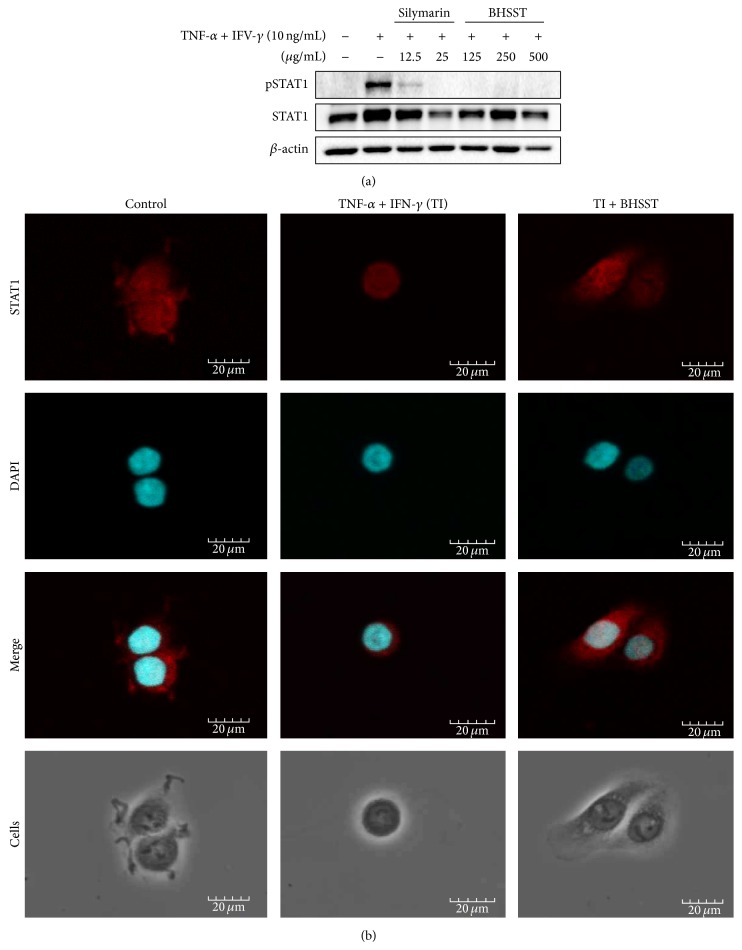
Effect of* Banhasasim-tang* (BHSST) on TNF-*α* and IFN-*γ*-induced STAT1 activation in HaCaT cells. (a) Expression of total and phosphorylated STAT1 was determined by Western blotting. Cells were cotreated with BHSST (125, 250, or 500 *μ*g/mL), and TNF-*α* and IFN-*γ* (TI, each 10 ng/mL) for 30 min. Silymarin (12.5 or 25 *μ*g/mL) was used as a positive control. Results are representative of at least three independent experiments. (b) Cellular localization of STAT1 was examined by immunofluorescence staining. Cells were cotreated with BHSST (500 *μ*g/mL), and TI for 30 min. The cells were fixed with 4% (v/v) methanol-free formaldehyde solution (pH 7.4), stained with anti-STAT1 (red). The stained cells were mounted with medium containing DAPI (blue) and visualized under an Olympus FLUOVIEW FV 10i confocal microscope.

Previous studies have reported that STAT1 is an important regulator of TI-induced inflammatory and immune responses [11, 12]. Therefore, we examined the effect of BHSST on the phosphorylation of STAT1 in TI-treated HaCaT cells. As shown in Figure 8, phosphorylation of STAT1 was increased by TI treatment. BHSST clearly inhibited TI-induced phosphorylation of STAT1. Treatment of silymarin reduced STAT1 phosphorylation (Figure 8(a)). Consistently, immunofluorescent staining revealed that BHSST blocked TI-induced nuclear localization of STAT1 in HaCaT cells (Figure 8(b)).

## 4. Discussion

BHSST, a traditional Korean herbal formula, has been used for the treatment of acute or chronic gastritis, diarrhea, and gastric ulcer [9, 10]. BHSST consists of eight medicinal herbs, Pinelliae Tuber, Scutellariae Radix, Ginseng Radix, Glycyrrhizae Radix et Rhizoma, Zingiberis Rhizoma, Coptidis Rhizoma, Zingiberis Rhizoma Crudus, and Zizyphi Fructus in 4 : 3 : 3 : 3 : 2 : 1 : 2 : 2 proportions. The main constituents of each herbal medicine are known as follows: phenolic acid (e.g., homogentisic acid) and phenolic aldehyde (e.g., 3,4-dihydroxybenzaldehyde) from Pinelliae Tuber [13], flavonoids (e.g., baicalin, wogonoside, baicalein, and wogonin) from Scutellariae Radix [14], triterpene saponins (e.g., ginsenoside Rb1) from Ginseng Radix [15], triterpene saponin (e.g., glycyrrhizin) and flavonoids (e.g., liquiritin and liquiritigenin) from Glycyrrhizae Radix et Rhizoma [16], phenols (e.g., 6-, 8-, and 10-gingerol) from Zingiberis Rhizoma and Zingiberis Rhizoma Crudus [17], alkaloids (e.g., berberine, coptisine, and palmatine) from Coptidis Rhizoma [18], and flavonoids (e.g., spinosin and 6′′′-feruloylspinosin) from Zizyphi Fructus [19].

Among those components, we analyzed nine compounds including baicalin, wogonoside, baicalein, and wogonin (Scutellariae Radix), glycyrrhizin and liquiritin (Glycyrrhizae et Rhizoma), and berberine, coptisine, and palmatine (Coptidis Rhizoma) using HPLC–PDA. An established HPLC–PDA method was applied for simultaneous analysis of the nine compounds in the BHSST sample. Baicalin (48.340 mg/g), a marker compound of Scutellariae Radix, was detected as the main component alongside other in the sample.

Previous studies demonstrated that* Pinellia ternate *[20],* Scutellaria baicalensis *[21],* Panax ginseng *[22],* Glycyrrhiza uralensis *[23],* Zingiber officinale *[24],* Coptis japonica* [25], and* Ziziphus jujuba* [26, 27] exert the anti-inflammatory and anti-AD effects. In addition, baicalin [28], wogonoside [29], baicalein [30], wogonin [31], and glycyrrhizae [32] have also been reported to exhibit anti-inflammatory and anti-AD effects. In particular, baicalin, the most abundant component in BHSST has been reported that the inhibitory effect on inflammatory mediators, cytokines, and TLR4/NF-*κ*B pathway activation [33]. Based on previous studies, we predicted that BHSST, containing these bioactive herbs as constituents, would exert beneficial effects on skin inflammatory diseases such as AD. Therefore, we investigated the anti-inflammatory effect of BHSST using LPS-stimulated RAW 264.7 macrophages and TI-stimulated HaCaT keratinocytes* in vitro*.

Inflammatory responses play a central role in the pathogenesis of many diseases, initiated by the invasion of pathogens or by tissue injury, followed by a series of vascular and cellular reactions. Macrophages are principal immune cells, which are activated by inflammatory mediators including NO and PGE_2_, as well as inflammatory cytokines such as TNF-*α*, IL-6, and IL-1*β* in inflammatory responses. High levels of these inflammatory mediators and cytokines in states of chronic inflammation can result in various pathological conditions [3, 4]. Therefore, these inflammatory mediators and cytokines are important targets for treating inflammatory diseases. In the present study, we observed that LPS stimulated the production of NO, PGE_2_, TNF-*α*, and IL-6 as well as expression of COX-2 in RAW 264.7 cells. By contrast, BHSST markedly reduced the production of NO, PGE_2_, and IL-6 in LPS-stimulated RAW 264.7 cells (Figure 4). These findings demonstrate that BHSST possesses anti-inflammatory effects by inhibiting production of NO, PGE_2_, and IL-6, whereas it is not controlled by TNF-*α* production and COX-2 expression in macrophages. Although BHSST had the inhibitory effect on PGE_2_ production, it did not affect COX-2 expression level. However, it may be possible that COX-2 enzymatic activity, but not expression, is involved in anti-inflammatory action of BHSST.

LPS activates the TLR4-mediated signaling pathway, and leading to the activation of NF-*κ*B to regulate the release of cytokines [34, 35]. To detect the inhibitory mechanism on inflammatory action of BHSST, we carried out the effect of BHSST on TLR4 and phosphorylation of NF-*κ*B. The results showed that BHSST pretreatment significantly suppressed LPS-stimulated TLR4 and NF-*κ*B p65 phosphorylation in RAW 264.7 cells (Figures 5 and 6). These finding suggested that BHSST exerts anti-inflammatory effect through the suppression of TLR4/NF-*κ*B pathway. It seems to be related to inhibitory effect of bioactive components of BHSST, such as baicalin [33].

Inflammatory mediators and cytokines aggravate inflammatory responses to skin lesions, which are an important feature of inflammation skin diseases such as AD [5]. Skin inflammation including AD develops on allergic reaction and is characterized by the overexpression of inflammatory cytokines. Furthermore, AD changed the expression of various inflammatory chemokines [36]. Chemokines are basic proteins that are secreted and critically important in allergic inflammation via leukocyte trafficking and activation. In particular, it has been reported that the serum level of RANTES, TARC, and MDC, which are Th2-type chemokines, is associated with skin inflammation [5, 6]. Moreover, IL-8, a member of the chemokine family, is produced by various types of cells upon stimulation with inflammatory stimuli [7]. Therefore, we investigated the effect of BHSST on RANTES, TARC, MDC, and IL-8 levels in TI-stimulated HaCaT cells, and found that BHSST inhibited the production of RANTES, TARC, MDC, and IL-8 in the cells (Figure 7). These findings suggest that BHSST regulates the recruitment of Th2-type cells into inflammatory skin lesions by suppressing production of inflammatory chemokines.

IFN-*γ* activates an inflammatory transcriptional program through the canonical Janus-kinase-signal transducer and activator of transcription (JAK-STAT) signaling pathway [12]. TARC production from TI-stimulated HaCaT cells is decreased by treatment with JAK/STAT inhibitors [11]. To confirm the mechanism of action of BHSST, we examined the effect of BHSST on STAT1 activation by TI stimulation in HaCaT cells. BHSST suppressed STAT1 phosphorylation and nuclear translocalization in TI-stimulated HaCaT cells (Figure 8).

In summary, our results demonstrate that BHSST exerts anti-inflammatory effects by reducing the production of NO, PGE_2_, and IL-6, as well as TLR4 and the phosphorylation of NF-*κ*B p65 in LPS-stimulated RAW 264.7 cells. BHSST exerts suppressive effects on the production of inflammatory chemokines RANTES, TARC, MDC, and IL-8, and the phosphorylation of STAT1 in TI-stimulated HaCaT cells. Overall, these findings provide evidence that BHSST can act as a preventive and therapeutic agent for inflammation-related skin diseases.

## Figures and Tables

**Table 1:** Composition of *Banhasasim-tang*.

Latin name	Scientific name	Amount (g)	Origin
Pinelliae Tuber	*Pinellia ternata *	7.500	China
Scutellariae Radix	*Scutellaria baicalensis *	5.625	Gurye, Korea
Ginseng Radix	*Panax ginseng *	5.625	Yeongju, Korea
Glycyrrhizae Radix et Rhizoma	*Glycyrrhiza uralensis *	5.625	China
Zingiberis Rhizoma	*Zingiber officinale *	3.750	Taean, Korea
Coptidis Rhizoma	*Coptis japonica *	1.875	China
Zingiberis Rhizoma Crudus	*Zingiber officinale *	3.750	Ulsan, Korea
Zizyphi Fructus	*Ziziphus jujuba *	3.750	Yeongcheon, Korea

Total		37.50	

**Table 2:** Contents of the nine marker components in the *Banhasasim-tang* by HPLC (*n* = 3).

Compound	Mean (mg/g)	SD	RSD (%)	Source
Liquiritin	5.412	0.014	0.253	*G. uralensis *
Coptisine	2.222	0.009	0.424	*C. japonica *
Baicalin	48.340	0.253	0.524	*S. baicalensis *
Palmatine	1.649	0.006	0.363	*C. japonica *
Berberine	3.909	0.001	0.038	*C. japonica *
Wogonoside	15.858	0.032	0.204	*S. baicalensis *
Baicalein	0.977	0.008	0.782	*S. baicalensis *
Glycyrrhizin	5.347	0.016	0.300	*G. uralensis *
Wogonin	1.087	0.002	0.145	*S. baicalensis *
